# Rapid generation of conditional knockout mice using the CRISPR-Cas9 system and electroporation for neuroscience research

**DOI:** 10.1186/s13041-021-00859-7

**Published:** 2021-09-23

**Authors:** Hirofumi Nishizono, Yuki Hayano, Yoshihisa Nakahata, Yasuhito Ishigaki, Ryohei Yasuda

**Affiliations:** 1 Medical Research Institute, Kanazawa Medical University, 1-1 Daigaku, Uchinada, Ishikawa 920-0293 Japan; 2 Max Planck Florida Institute for Neuroscience, 1 Max Planck Way, Jupiter, FL 33458 USA

**Keywords:** Cre/LoxP, floxed mouse, CRISPR-Cas9, genome editing, CaMK1

## Abstract

The Cre/LoxP-based conditional knockout technology is a powerful tool for gene function analysis that allows region- and time-specific gene manipulation. However, inserting a pair of LoxP cassettes to generate conditional knockout can be technically challenging and thus time- and resource-consuming. This study proposes an efficient, low-cost method to generate floxed mice using in vitro fertilization and the CRISPR-Cas9 system over two consecutive generations. This method allowed us to produce floxed mice targeting exons 5 and 6 of *CaMK1* in a short period of 125 days, using only 16 mice. In addition, we directly edited the genome of fertilized eggs of mice with our target genetic background, C57BL/6 N, to eliminate additional backcrossing steps. We confirmed that the genome of the generated floxed mice was responsive to the Cre protein. This low-cost, time-saving method for generating conditional knockout will facilitate comprehensive, tissue-specific genome analyses.

The invention of Embryonic stem (ES) cell-based gene targeting technology in mice and conditional knockout systems using Cre/LoxP has led to breakthroughs in neuroscience [1]. These technologies have allowed the analysis of gene function in a region- or time-specific manner. However, they required a long time and expensive equipment [2, 3]. Moreover, microinjection requires highly skilled personnel and expensive equipment; additionally, this technique has a low success rate. Recently, the development of genome editing technology for fertilized eggs combined with electroporation and the CRISPR-Cas9 system (TAKE and iGONAD) has made it possible to generate gene-modified mice easily and with a high success rate using inexpensive equipment [4–6]. However, electroporation permits only short (1 kb or less) single-stranded DNA (ssDNA) to be introduced into the nucleus [7]. Thus, while the electroporation-based method allows for the efficient development of the knock-in of a relatively short tag, it is not suitable for creating conditional knockout mice for the deletion of a long genomic region, which can be performed by a LoxP-multiple exons-LoxP cassette. To overcome this limitation, a research group recently introduced the Easi-CRISPR method that can efficiently insert a relatively long transgene into the genome of fertilized eggs using long ssDNA and CRISPR ribonucleoproteins [8]. However, this approach does not allow the generation of floxed mice targeting regions larger than 1.5 kb. Furthermore, long ssDNA is often challenging and expensive to synthesize. Other research groups have reported that it is possible to introduce LoxP sequences on the 5’ and 3’ sides by two sequential electroporation during embryonic development following fertilization [9]. This method could theoretically be used even if the region to be knocked out is larger than 1.5 kb using only electroporation. However, it has been difficult to reproduce this method in large-scale studies with multiple experimental facilities [10].

As a compromise between the simplicity and the time needed for generating conditional knockout mice targeting regions larger than 1.5 kb, we attempted to generate conditional knockout mice within two generations (Fig. 1A, B). Exons 5 and 6 of the Ca^2+^/calmodulin-dependent protein kinase 1 gene (*CaMK1*) [11] were targeted to be knocked out under Cre recombinase expression. Briefly, a DNA donor containing LoxP and a homology arm (5’ LoxP) was knocked into the upstream intron of exon 5. A similar short construct was inserted into the downstream intron of exon 6 (3’ LoxP). These two DNA donors were single-stranded oligo donors (ssODNs) of less than 200 bases each; thus, they could be transferred into the pronucleus of the fertilized eggs by electroporation [12]. First, we knocked in 5’ LoxP into C57BL/6 N mouse embryos by electroporation with gRNA and Cas9 protein and then transferred the embryos to pseudopregnant females. When the resulting 5’ LoxP-bearing mice were 6–8 weeks old, we performed a second round of *in vitro* fertilization (IVF) using their sperm and wild-type oocytes. Fertilized eggs carrying 5’ LoxP were electroporated with the 3’ LoxP ssODN. Subsequently, four mice carrying both 5’ and 3’ LoxP were born among ten mice in the second round of IVF. The results of long PCR analysis (Fig. 1C) using PCR primers comprising the LoxP sequences (Fig. 1A) indicated that both LoxPs were introduced into the same allele in one mouse. Furthermore, sequencing results indicated that each LoxP was in the correct position and direction (Fig. 1D).

**Fig. 1:**
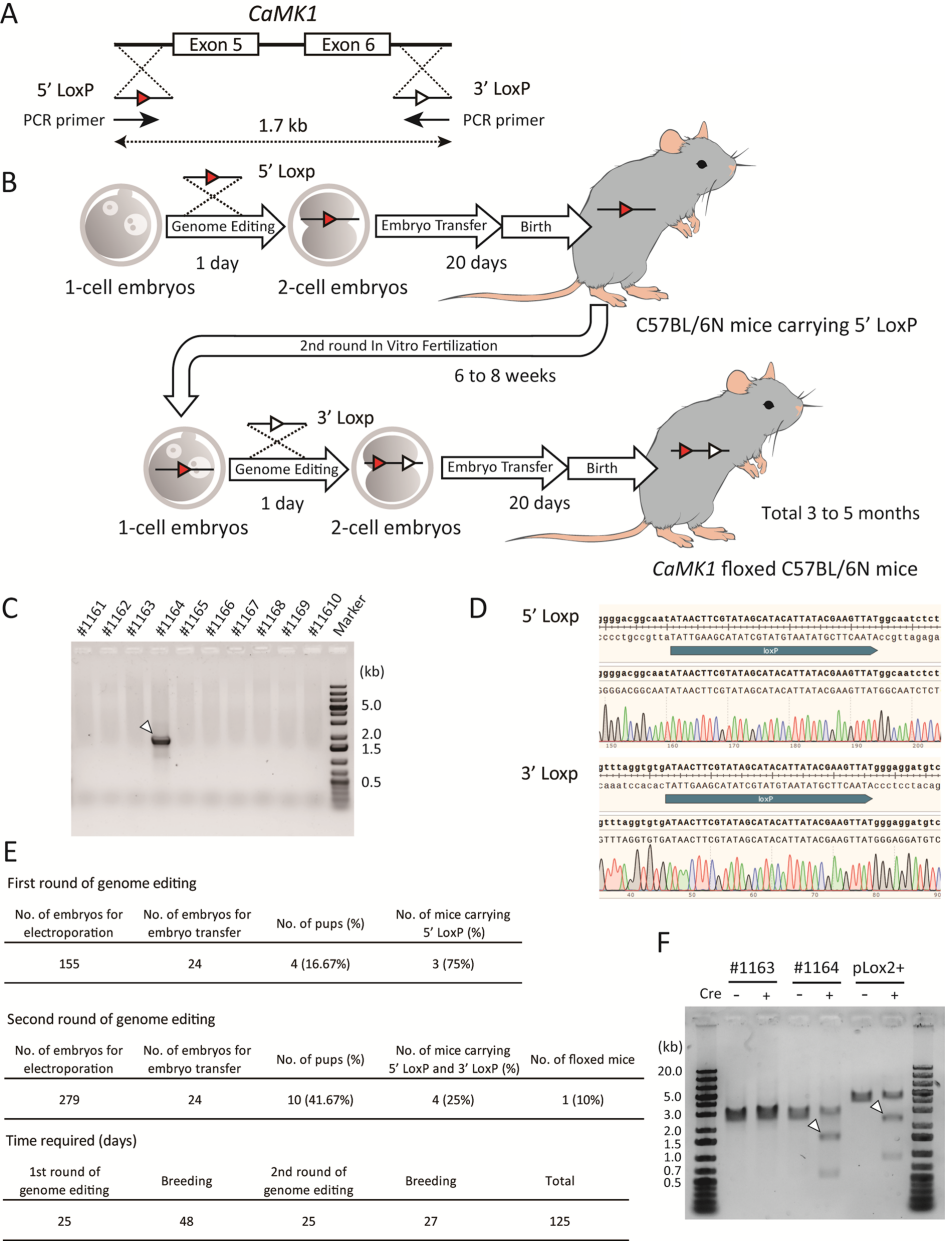
Generation of *CaMK1* floxed mice. **A** Diagram of the constructs. The gRNAs and the ssODNs were designed to introduce a 5’-side LoxP (5’ LoxP) into the intron upstream of exon 5, and a 3’-side LoxP (3’ LoxP) into the intron downstream of exon 6. The distance between the LoxPs is 1.7 kb. **B** Scheme of sequential in vitro fertilization and genome editing over two generations. **C** Genotyping PCR of ten mice born after two rounds of genome editing. Arrowheads indicate target bands. **D** DNA sequencing around the LoxPs of *CaMK1* floxed mouse (#1164). The upper panel is around 5’ LoxP, and the lower panel is around 3’ LoxP. No deletion or mismatch could be identified. **E** Detailed results of the first and second rounds of genome editing. **F** in vitro Cre protein treatment of the PCR product from *CaMK1* floxed mouse (#1164) genome. pLox2+, a 3.6 kb plasmid with two LoxPs, was used as a positive control. A littermate (#1163) with two LoxPs on different alleles was used as a negative control. Arrowheads indicate the excised DNA. The expected size of the excised DNA is 2787 bp for pLox2+, and the rest is 838 bp. In *CaMK1* floxed mice, the expected size of the excised DNA is 1772 bp, and the rest is 527 bp. +; Cre protein-treated group, −; group not treated with Cre protein

The efficiency of this method for generating floxed mice is shown in Fig. 1E. These results indicate that each genome editing treatment efficiently introduced ssODNs into the genome. After the first and second rounds of electroporation, the embryo survival rates were 97.42  and 98.21 %, respectively. Embryos that developed to the 2-cell stage on the day after electroporation were randomly selected and transferred to the oviduct. Electroporation had no significant effect on embryonic development or implantation. We tested the simultaneous insertion of two LoxPs into embryos using the same ssODNs and gRNAs in a single IVF round [9]. However, even after two cycles, no mice were born after embryo transfer. The results were the same as those obtained using other ssODNs and gRNAs. Recently, Shang et al. published a method to introduce two LoxPs using iGONAD simultaneously [13]. This method is expected to be a powerful tool. However, two concerns remain with this method. The birth rate decreased in the C57BL/6 background mice because it is an iGONAD-based method. There is a possibility of genetic loss because two gRNAs are introduced simultaneously. Therefore, the reproducibility of this method needs to be confirmed. Our method has none of these problems.

We succeeded in creating a new floxed line with fewer than 20 C57BL/6 N mice using this method. Theoretically, the time required could be as short as three months, but it took 125 days in practice. Thus, it is possible to produce the desired floxed mice in a markedly short period. Takeo and Nakagata reported that the use of anti-inhibin serum for superovulation makes it possible to use 4-week-old female mice for IVF [14]. Therefore, applying this technique to the second round of IVF could shorten the time further. To examine whether the two introduced LoxPs were functional, PCR products containing the region between LoxPs of *CaMK1* floxed mice were treated with recombinant Cre protein in a cell-free assay, using a previously described method [15]. In the presence of Cre protein, the excised bands were identified in the positive control, pLox2+ (a 3.6 kb plasmid in which two LoxPs are introduced), and in the PCR product of *CaMK1* floxed mice (#1164), as shown in Fig. 1F. In contrast, no truncated bands were observed in the samples from a littermate (#1163) with 5’ LoxP and 3’ LoxP on different alleles. These results indicate that the generated *CaMK1* floxed mice can become a conditionally knockout using Cre driver mice or other methods. Nonetheless, further validation with other genes is required. We genome-edited the fertilized eggs of mice with our target genetic background C57BL/6 N. Thus, no backcrossing was required, in contrast to the ES cell-based method.

## Materials and methods

### Genome editing

Genome editing was performed using the method described in our previous study [13]. Briefly, the crRNA and tracrRNA were mixed and annealed at 95 °C for 3 min to prepare the gRNA. Within 5 h after in vitro fertilization, the 1-cell stage embryos of C57BL/6 N mice (6–8 weeks old, Charles River, Wilmington, MA, USA) were introduced into electroporation solution, which was prepared with 100 ng/µL gRNA, 20 ng/µL ssODN, and 100 ng/µL HiFi Cas9 in a nuclease-free duplex buffer. We used a NEPA21 electroporator (Nepa Gene Co., Ltd., Chiba, Japan). The following day, only 2-cell embryos were transferred into the oviducts of pseudopregnant females. Two crRNAs (5’- tatgcaccaggggacggcaatgg-3’ for 5’ LoxP and 5’- ggtgtgatccggtttaggtgtgg − 3’ for 3’ LoxP) and one tracrRNA were used. Two types of ssODNs, 5’-ctgcacgacctgggcattgtgcaccgggatctcaaggtaggatctgaggggcctagtgaactatatgcaccaggggacggcaatATAACTTCGTATAGCATACATTATACGAAGTTATggcaatctctgtctgtcctgctttgtctgtctttgagtacctctcagcccctcactaaagccctagctttccatttgcaa-3’ and 5’-tcacttcagatagtcaaaggccctttgtgatggtaaaatctgagtggcttttgagccagtttaggtgtgatccggtttaggtgtgATAACTTCGTATAGCATACATTATACGAAGTTATgggaggatgtcaaacatgaagaccctatgacagcatgttcaaggacagaaggaaggccagtactgccagacagaagtgag-3’, were used as a transgene. We used 5’-acattatacgaagttatggcaatct-3’ and 5’-gctatacgaagttatcacacctaaacc-3’ primers for genotyping PCR. All DNA and RNA oligos and reagents were purchased from Integrated DNA Technologies, Inc. (Coralville, IA, USA).

### Cre protein treatment

In vitro Cre protein treatment was performed as previously described by Zhang et al. [15]. We used the DNA fragments amplified from the *CaMK1* floxed mouse genome by long PCR using the primers 5’-ggtttcaggtggagagctgt-3’ and 5’-cagagtcagagatgtcgtccca-3’. The purified PCR products of *CaMK1* floxed mouse genome and control plasmid (pLox2+, M0298, New England Biolabs, Ipswich, MA, USA) were incubated at 37 °C for 30 min with or without Cre protein. DNA cleavage was confirmed using electrophoresis.

## Data Availability

The authors declare that all data supporting the findings of this study are available within the article.
